# Supplementary material for: The Antivirulence Activity of Umbelliferone and Its Protective Effect against A. hydrophila-Infected Grass Carp
Source: Int J Mol Sci. 2022 Sep 22;23(19):11119. doi: 10.3390/ijms231911119 (PMC9569924; doi:10.3390/ijms231911119)
Supplement: Supplementary file 1 [file ijms-23-11119-s001.zip › Supplementary Table S1 sequencing data summary.pdf]

Table S1. Overview of sequencing data

Sample name	Raw reads	Clean reads	clean bases	Mapped ratio (%)	Q30(%)
C1	7569420	7540054	1.1G	93.80%	95.44
C2	7997372	7944600	1.2G	94%	95.41
C3	7749420	7697060	1.2G	94.83	95.16
T1	7575978	7521680	1.1G	94.93	95.03
T2	8324810	8246466	1.2G	95.38	95.05
T3	7883550	7820140	1.2G	95.58	95.41
