# Supplementary material for: The Antivirulence Activity of Umbelliferone and Its Protective Effect against A. hydrophila-Infected Grass Carp
Source: Int J Mol Sci. 2022 Sep 22;23(19):11119. doi: 10.3390/ijms231911119 (PMC9569924; doi:10.3390/ijms231911119)
Supplement: Supplementary file 1 [file ijms-23-11119-s001.zip › Supplementary Table S4 qPCR primer.pdf]

Table S4. qPCR primers

Primer		Sequence (5′-3′)	PCR product length (bp)
tssK	F:	AGGATTACCTGGGCTTTGGC	101
	R:	GGTCGGCAGCACATCGTCA	
exeN	F:	GCTGATTGCCGTGCTGTTTC	169
	R:	CGACTCGCTGGCGTATTGC	
ampC	F:	ATGAAACAGACCAGAGCCTTGC	199
	R:	CCAGCCCGTAGTTGAAGTAGTG	
tsgA	F:	CACCTACGGTTACGGCTTTG	96
	R:	GAGACGTTCTTGCTCGATGC	
vgrG	F:	CAGTCTTGGCAACGATCATCT	80
	R:	TATCCACGGTCAGCGAGTAG	
emrD	F:	CGCCTTGGCATCGTCTTC	144
	R:	GGAGGTAGCGGTCGGATAGA	
pspG	F:	GGCGTCTTCGTGCTGTT	130
	R:	GGTAGGTATTGGCCTGCTT	
amoA	F:	ACTACCCCTATGCCACCCG	164
	R:	GTCGTGCCAGTCGTCATCG	
apoA	F:	GCTGGAAGGCAAGGACGAGGTAAC	205
	R:	AACACGAGCAGAAGCAGGCACATAA	
